# Mapping *Histoplasma capsulatum* Exposure, United States

**DOI:** 10.3201/eid2410.180032

**Published:** 2018-10

**Authors:** Amelia W. Maiga, Stephen Deppen, Beth Koontz Scaffidi, John Baddley, Melinda C. Aldrich, Robert S. Dittus, Eric L. Grogan

**Affiliations:** Vanderbilt University Medical Center, Nashville, Tennessee, USA (A.W. Maiga, S. Deppen, M.C. Aldrich, R.S. Dittus, E.L. Grogan);; Tennessee Valley Healthcare System Veterans Hospital, Nashville (A.W. Maiga, R.S. Dittus); Vanderbilt University, Nashville (B.K. Scaffidi);; University of Alabama at Birmingham, Birmingham, Alabama, USA (J. Baddley)

**Keywords:** endemic mycoses, histoplasmosis, mapping, suitability score, *Histoplasma capsulatum*, fungi, United States

## Abstract

Maps of *Histoplasma capsulatum* infection prevalence were created 50 years ago; since then, the environment, climate, and anthropogenic land use have changed drastically. Recent outbreaks of acute disease in Montana and Nebraska, USA, suggest shifts in geographic distribution, necessitating updated prevalence maps. To create a weighted overlay geographic suitability model for *Histoplasma*, we used a geographic information system to combine satellite imagery integrating land cover use (70%), distance to water (20%), and soil pH (10%). We used logistic regression modeling to compare our map with state-level histoplasmosis incidence data from a 5% sample from the Centers for Medicare and Medicaid Services. When compared with the state-based Centers data, the predictive accuracy of the suitability score–predicted states with high and mid-to-high histoplasmosis incidence was moderate. Preferred soil environments for *Histoplasma* have migrated into the upper Missouri River basin. Suitability score mapping may be applicable to other geographically specific infectious vectors.

Histoplasmosis is a regionally endemic mycosis caused by inhalation of the spores of the fungus *Histoplasma capsulatum* from the soil, leading occasionally to symptomatic disease and asymptomatic pulmonary nodules (*1*). Soils act as reservoirs for *Histoplasma*, especially where temperatures are 22°C–29°C and annual rainfall is 35–50 inches (*2*). According to a 1969 map by Edwards et al. of histoplasmin skin test reactivity among single-county-resident Navy recruits in the United States, *H. capsulatum* has been most prevalent in the Mississippi and Ohio River basins (*3*). Since then, much has changed with the environment, climate, and anthropogenic land use. Indeed, the advent of HIV/AIDS and the use of immunosuppressive medications for rheumatologic, dermatologic, and gastrointestinal conditions has unmasked areas of previously hidden histoplasmosis endemicity, nationally and globally (*4*). Recent outbreaks in Montana and Nebraska have led the Centers for Disease Control and Prevention to suggest that histoplasmosis is now endemic to these regions (*5*,*6*). State-level incidence rates of histoplasmosis among older patients also demonstrate a shift toward Nebraska and the northern Great Lakes area (*7*). In addition, a recent publication of surveillance data from 2011 through 2014 in 12 states suggests that histoplasmosis also occurs in Minnesota, Wisconsin, and Michigan, areas where histoplasmosis was not thought to be endemic (*8*).

Accurate maps of *H. capsulatum* prevalence assist in the early recognition, diagnosis, and treatment of acute infections. Pulmonary histoplasmosis may generate cancer-mimicking lung granulomas, causing false-positive radiographic images on high-resolution computed tomography and fluorodeoxyglycose positron emission tomography (FDG-PET) (*9**–**11*). Thus, an accurate understanding of the conditions for *H. capsulatum* is of practical epidemiologic and clinical value.

Edwards’ 1969 survey of histoplasmosis endemicity is impractical to repeat now or in the foreseeable future (*3*). It would be prohibitively expensive to survey a sufficiently large sample of the population and, in our mobile society, nearly impossible to identify an appropriate number of lifetime-single-county residents who are representative of the broader population. Furthermore, the histoplasmin skin test is no longer available, and another approach is needed. Our study objective was to model known environmental factors preferred by *H. capsulatum* and produce a large-scale map to enable risk stratification of patients on the basis of their geographic history.

## Materials and Methods

We created a site suitability model for *H.* *capsulatum* weighted by 3 environmental soil characteristics measured across the continental United States within a geographic information system (ArcGIS 10.3.1; ESRI, Redlands, CA, USA). Suitability scores ranged from 1 through 9 within each soil characteristic; higher scores signified more favorable conditions for *H. capsulatum*. Selection of raster datasets was based on known criteria for histoplasmosis behavior—land cover, water, and soil pH—because cultivation and anthropogenic land use increase the spread of *H. capsulatum* from the soil to the air (*1*).

### Land Cover

We obtained land cover data from the 2006 National Land Cover Database (*12*). This publicly available dataset applies 16 classes of land cover or land use types over the conterminous United States at a spatial resolution of 30 × 30 meters. The risk for histoplasmosis growth is highest in areas of high soil humidity (e.g., near rivers), and soil moisture affects the temperature at which *H. capsulatum* can survive (*13**–**15*).

### Distance from Water

Because no continuous flood risk map exists for the United States, we defined water presence by attributes extracted from the 2006 National Land Cover Database (e.g., open water, woody wetlands, or emergent herbaceous woodlands). Suitability for *H. capsulatum* was modeled to decrease with Euclidean distance from these water types across 9 equally distributed categories. Suitability scores ranged from a best-suited value of 9, which occurred from 0 through 222 meters, to a low value of 1, at >1,777 meters (Table). Open water was assumed to preclude presence of histoplasmosis.

**Table:** Scoring of 3 soil characteristics used for *Histoplasma* site suitability map*

Assigned value	Land cover class (70% weight)†	Meters from water (20% weight)	Soil pH (10% weight)
9	Cultivated crops, >20% vegetation	0–222	7.2–7.6
8	Pasture or hay, >20% vegetation	222–444	7.0–7.2 or 7.6–7.8
7	Open water, woody wetlands, >20% vegetation; or emergent herbaceous wetlands, >80% vegetation	444–666	6.7–7
6	Deciduous, evergreen or mixed forest, >20% vegetation	666–888	6.4–6.7 or 7.8–8.0
5	Dwarf scrub or shrub/scrub, >20% vegetation; or grassland used for grazing, >80% vegetation	888–1,110	6.0–6.4
4	Developed, open space such as lawns, <20% impervious	1,110–1,332	5.6–6.0 or >8
3	Developed, low and medium intensity, 20% to 79% impervious	1,332–1,555	5.1–5.6
2	Barren land such as rock, sand, or clay, <15% vegetation	1,555–1,777	>4.5 and <5.1
1	Developed, high intensity, >80% impervious	1,777–1,999	<4.5

*A value of 9 represents the most suitable environment for *H. capsulatum*. The overall weighted score was calculated as follows: an area of evergreen forest, located 1,000 meters from water, with a soil pH of 7.7 would have a suitability score of (6 × 0.7) + (5 × 0.2) + (8 × 0.1) = 6. †Excluded classes include perennial ice/snow and Alaska-only vegetation types.

### Soil pH

We obtained soil pH raster data from the University of Wisconsin Atlas of the Biosphere, compiled by the International Geosphere-Biosphere Programme Global Soils Data Task in 1998 (*16*). Original data projection of the Environmental Systems Research Institute ArcView gridded format with a spatial resolution of 10,000 meters, or 10 km, was reclassified into 9 categories. The highest score of 9 was assigned to pH values near 7.4, the pH of normal lungs, the presumed ideal environment for the *H. capsulatum* exposure of interest. Decreasing pH values were modeled in a descending fashion to a score of 4 for pH >8 and a score of 1 for pH <4.5 (Table).

### Mapping

We calculated the overall score for each ZIP code area as a weighted sum of assigned values from each of the 3 environmental factors. Each layer was preprocessed for analysis by mosaicking native coordinate systems across a common geographic coordinate system (World Geodetic System 1984, https://www.nga.mil/ProductsServices/GeodesyandGeophysics/Pages/WorldGeodeticSystem.aspx) and then clipping the data for each raster layer to the conterminous US border. Pixels for each layer were reclassified to a common scale of 1–9, ranging from least to most suitable for *H. capsulatum*. The unique map layers were then weighted and combined. Weighting was an iterative process to approximate the historical distribution of *Histoplasma* and recent histoplasmosis infection patterns, especially in the lower Mississippi River valley and, given the limitations of computational efficiency, to interpolate the final risk surface (*17*). The final suitability model included 3 layers of data: the 2006 National Land Cover Database with 16 classes of land cover or land use types (weighted 70%), Euclidean distance from the nearest open water source (20%), and soil pH (10%) (Table). A granular risk score was computed for each pixel. Next, the pixel values from the suitability map were extracted as points, and those points were joined to state and census tract polygons to provide a mean, median, and SD for exposure suitability scores aggregated to each spatial unit. To create a continuous exposure risk surface, we applied the inverse distance weights to interpolate a risk surface, which was then clipped to the US border.

We determined the 70%, 20%, and 10% weights through an iterative process based on Edwards’ 1969 survey county-level data for a few states (Tennessee with high within-state variability, Mississippi and Kansas with low variability, and Florida as a coastal state). During the developmental phase of the suitability score, we compared different proportions with state-level Medicare data, but there were no better models. Because of the lack of granularity of the state data, our sensitivity analyses may not have been very informative for detecting minor differences in state prevalence.

### Statistical Analyses

We summarized our data to the state level to permit comparison with previously published research (*7*). The reference standard was state-level histoplasmosis incidence data from a random 5% sample of the Centers for Medicare and Medicaid Services (CMS) claims data for 1999–2008 (*7*). Incident cases of endemic histoplasmosis required 1 inpatient claim or 2 outpatient claims over 7–90 days. We used 2 logistic regression models to assess the ability of our suitability histoplasmosis score to predict the binary outcome of correctly identifying states with high histoplasmosis incidence rates, defined as >5 cases/100,000 person-years, and states with mid-to-high incidence rates, defined as >3 cases/100,000 person-years. We generated the appropriate area under the receiver operator curves (AUCs) and calculated the Wilcoxon-Mann-Whitney test by using Stata version 12.0 (StataCorp LLC, College Station, TX, USA).

## Results

**Figure 1:**
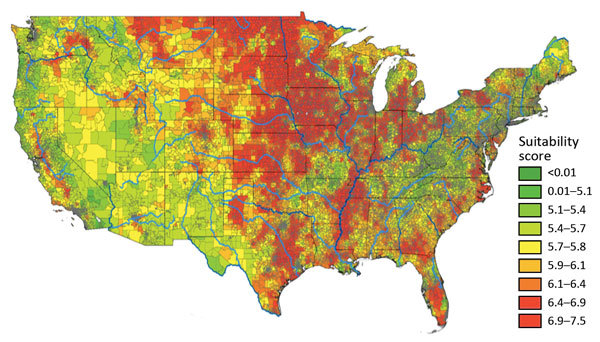
Mean *Histoplasma* site suitability score by US ZIP code. Red reflects greater histoplasmosis suitability; green reflects less suitability. The weighted mean score (Table) was calculated for each ZIP code. Data for geographic regions west of the Rocky Mountains are considered insufficient because of limited surface water data.

**Figure 2:**
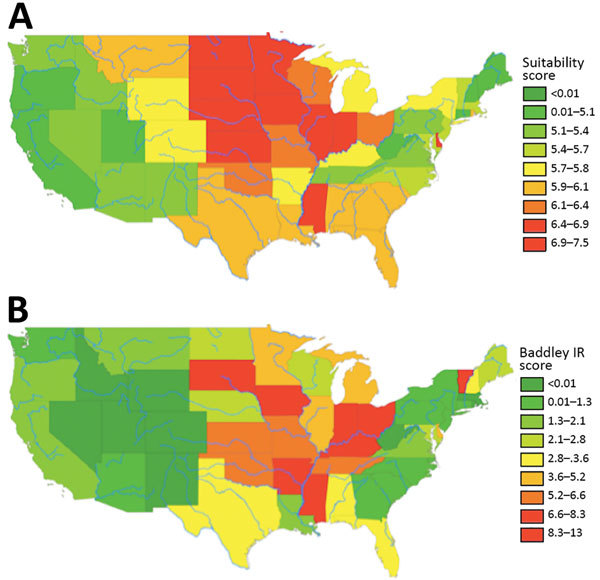
State-level suitability score compared with histoplasmosis incidence rates, United States. A) State-level suitability score map. B) State-level histoplasmosis incidence rates for 1999–2008 US Medicare and Medicaid data (no. cases/100,000 person-years).

Figure 1 depicts the granular suitability score map with mean scores by US ZIP code. The data and model are applicable to estimates of *Histoplasma* in soils east of the Rocky Mountains because of lack of suitable soil habitats and limits in data availability. We calculated suitability score estimates west of the Rocky Mountains, but many geographic blocks had no or limited water data and thus may overestimate *Histoplasma* suitability (e.g., distances >3,000 meters from substantial amounts of water may preclude the presence of *Histoplasma* irrespective of land use). Figure 2 compares the state-level suitability scores with 1999–2008 state-level incidence data from Baddley et al. (*7*). Our suitability score model identified the states with high and mid-to-high histoplasmosis incidence rates with AUCs of 0.72 and 0.74, respectively, when compared with state-based diagnosis rates among elderly persons during 1999–2008.

## Discussion

Our histoplasmosis exposure suitability score predicted state-level histoplasmosis incidence rates from recent CMS data with an AUC >0.72. Our model confirms previous reports that preferred soil environments for *Histoplasma* have expanded into the upper Missouri River basin. This progression probably reflects changes in climate and human land use with increased cultivation in that region and urbanization of other regions of the country.

Our suitability score has limitations. We compared our score to state-level histoplasmosis incident cases as derived from administrative data and were thus unable to assess the validity of our score to predict disease incidence for a more granular geographic region. However, CMS is not likely to release county-level histoplasmosis infection rates because of the risk of identifying individual patients. The administrative data we used for our comparative analysis have their own limitations, namely reliance on codes from the International Classification of Disease, Ninth Revision for histoplasmosis rate estimates, with unknown sensitivity and specificity.

We also did not include known specific vectors of histoplasmosis exposure (e.g., droppings in areas of bird or bat roosts). This exclusion is particularly relevant, given the microfocus concept that *Histoplasma* exist in small environmental foci, resulting in localized outbreaks or other exposures in non–histoplasmosis-endemic areas (*17**,**18*). It is also worth noting that the coarse resolution of the soil pH pixels limited the sensitivity of the weighted overlay result. Local studies that use soil pH from US Department of Agriculture data within each state at a smaller resolution may be preferred to upsampling the coarse International Geosphere-Biosphere Programme data. The accuracy of our approach should be confirmed with soil pH measurements. In addition, as with any aggregate measure, our score is subject to the ecologic fallacy, whereby inferences about individual risk are estimated by an overall estimate from the geographic region in which the person resides. This suitability score could be combined with occupational and residential histories to risk-stratify patients for fungal lung disease risk and to improve interpretation of chest images.

The accurate diagnosis of histoplasmosis is challenging and often requires a battery of antigen detection, serology, and histopathology studies (*19**,**20*). Serologic tests are imperfect and, although often specific, can have low sensitivity, particularly for immunosuppressed patients. Knowledge of the distribution of *H. capsulatum* exposure and histoplasmosis infection is also critical for the appropriate regional use of imaging to diagnose fungal lung disease in immunocompromised persons and persons with lung malignancies. Investigators have questioned the utility of FDG-PET for diagnosing lung nodules in regions where granulomatous disease is prevalent (*9**,**10**,**21*). A recent meta-analysis concluded that FDG-PET was less specific for diagnosing malignancy in populations where lung mycosis is endemic than in those where it is not (*11*).

In conclusion, we generated a detailed suitability map for *Histoplasma* exposure east of the Rocky Mountains, an approach that can be updated as land use changes. Our suitability map may provide a detailed prediction of the risk for acute histoplasmosis infection, including areas of new endemicity. Accurate mapping of *Histoplamosis* exposure may also aid in the interpretation of chest images, particularly in the context of lung cancer screening programs (*22*). Future work might add elevation, soil moisture, water-holding capacity, and geologic data into the suitability model. Suitability score models have the potential to be applied to other infectious agents strongly associated with geographic-specific vectors, with the promise to inform healthcare and improve public health assessments and interventions.
